# Supplementary material for: The Construction and Exploration of a Comprehensive MicroRNA Centered Regulatory Network in Foxtail Millet (Setaria italica L.)
Source: Front Plant Sci. 2022 May 6;13:848474. doi: 10.3389/fpls.2022.848474 (PMC9121102; doi:10.3389/fpls.2022.848474)

T=Seita.5G438900.1_Q=Sit−miR169g_S=506

category=2_p=0.999552617553945

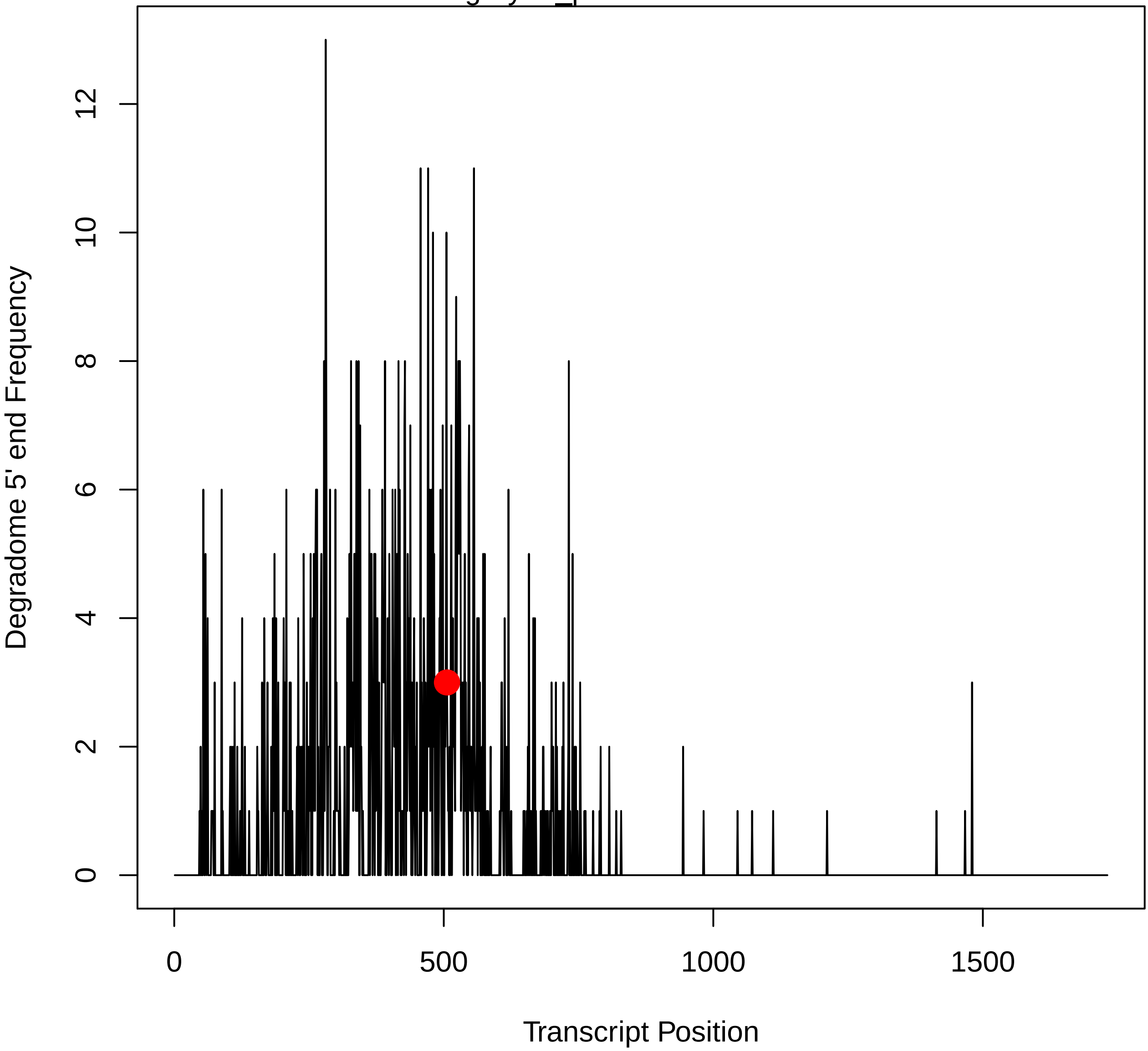

Supplement: Supplementary file 6 [file Data_Sheet_6.zip › Sit-miR169g_Seita.5G438900.1_506_TPlot.pdf]
